# Clinical Value of ^18^F-FDG PET in Paraneoplastic and Pembrolizumab-Associated Myositis

**DOI:** 10.3390/diagnostics16040596

**Published:** 2026-02-17

**Authors:** Chang-Shen Tseng, Yu-Hung Chen, Ching-Chun Ho, Kuei-Ying Su, Sung-Chao Chu, Shu-Hsin Liu

**Affiliations:** 1 Department of Nuclear Medicine, Hualien Tzu Chi Hospital, Buddhist Tzu Chi Medical Foundation, Hualien 970473, Taiwan; 104311113a@gmail.com (C.-S.T.); kaopectin@yahoo.com.tw (S.-H.L.); 2 School of Medicine, Tzu Chi University, Hualien 970374, Taiwan; oldguy-chu1129@umail.hinet.net; 3 Department of Medical Imaging and Radiological Sciences, Tzu Chi University, Hualien 970374, Taiwan; 4 Department of Surgery, Hualien Tzu Chi Hospital, Buddhist Tzu Chi Medical Foundation, Hualien 970374, Taiwan; natalie.hcc@gmail.com; 5 Division of Allergy, Immunology and Rheumatology, Hualien Tzu Chi Hospital, Buddhist Tzu Chi Medical Foundation, Hualien 970374, Taiwan; kueiying@gmail.com; 6 Department of Hematology and Oncology, Hualien Tzu Chi Hospital, Buddhist Tzu Chi Medical Foundation, Hualien 970374, Taiwan

**Keywords:** ^18^F-FDG PET, myositis, pembrolizumab

## Abstract

Fluorine-18 fluorodeoxyglucose positron emission tomography (^18^F-FDG PET) is widely used to evaluate patients with cancer and to detect various inflammatory processes. Here, we report a rare case of a 53-year-old woman with breast cancer who developed generalized pain, weakness, rash, and edema over the trunk after the first cycle of neoadjuvant pembrolizumab and cytotoxic chemotherapeutic agents. ^18^F-FDG PET revealed diffusely increased uptake in the muscles without skeletal or visceral metastasis, aiding in the diagnosis of paraneoplastic and pembrolizumab-associated myositis and subsequent monitoring. This case underscores the value of ^18^F-FDG PET in differentiating myositis from tumor progression and monitoring treatment response in such cases.

**Figure 1:**
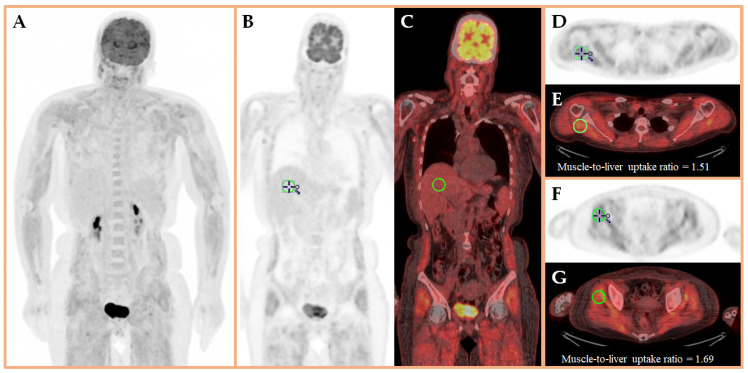
A 53-year-old woman with triple-negative breast cancer (TNBC), classified as cT2N1M0 [1], received neoadjuvant therapy with pembrolizumab and cytotoxic chemotherapeutic agents [2]. After the first cycle, she developed generalized pain, weakness, rash, and edema over the trunk and limbs, eventually becoming bedridden. Fluorine-18 fluorodeoxyglucose positron emission tomography (^18^F-FDG PET) was performed to rule out disease progression and skeletal metastasis. The patient fasted for at least 4 h before intravenous ^18^F-FDG injection (400.00 MBq). The patient did not exercise before the injection. We performed the ^18^F-FDG PET scan from head to thigh 60 min after the radiotracer injection. (**A**): Maximum Intensity Projection showing diffusely increased uptake in the muscles without skeletal or visceral metastasis. Coronal (**B**,**C**) and axial PET and fused PET/CT images showed the scapular (**D**,**E**) and gluteal (**F**,**G**) muscle-to-liver mean standardized uptake value (SUV_mean_) ratios of 1.51 and 1.69, respectively. Laboratory tests showed elevated creatine kinase at 1945 U/L (reference range: 30–223 U/L) [3] and positive anti-transcription intermediary factor 1-γ autoantibodies. Taken together, these findings confirmed the diagnosis of paraneoplastic and pembrolizumab-induced myositis [4,5,6].

**Figure 2:**
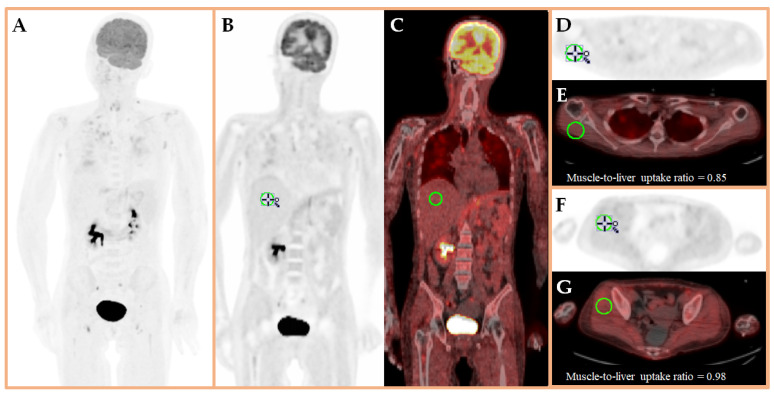
The paraneoplastic and pembrolizumab-induced myositis were treated with intravenous immunoglobulin (2 g/kg) and prednisolone at an equivalent daily dose of 50 mg [7,8,9]. The prednisolone dose was gradually tapered to 5 mg daily over a two-month period. The patient subsequently underwent a modified radical mastectomy, with a final pathological stage of ypT2N1a. Follow-up ^18^F-FDG PET performed 5 months after the initial scan revealed reduced muscular ^18^F-FDG uptake (**A**–**C**), with scapular (**D**,**E**) and gluteal (**F**,**G**) muscle-to-liver SUV_mean_ ratios of 0.85 and 0.98, respectively. The creatine kinase level normalized, and her symptoms gradually subsided. She could walk using a walker and required minimal assistance with self-care. Myositis is a rare comorbidity among patients with TNBC, whether as an immune-related adverse event of pembrolizumab or as a paraneoplastic manifestation. In this case, the onset occurred immediately after the first cycle of pembrolizumab, suggesting that the pembrolizumab may trigger a latent paraneoplastic phenomenon. While serum creatine kinase levels are routinely used as a biomarker of muscle injury, ^18^F-FDG PET enables direct visualization of inflammatory activities, allows assessment of myositis severity, and assists in distinguishing myositis from cancer metastasis. This case demonstrates the value of ^18^F-FDG PET as a molecular imaging modality to evaluate patients with cancer with suspected paraneoplastic or immune checkpoint inhibitor-associated myositis.

## Data Availability

The data presented in this study are available on request from the corresponding author due to ethical restrictions.

## Abbreviations

The following abbreviations are used in this manuscript:

FDG	Fluoro-deoxy-glucose
PET	Positron emission tomography
SUV_mean_	Mean standardized uptake value
TNBC	Triple-negative breast cancer
